# Does prolonged β-lactam infusions improve clinical outcomes compared to intermittent infusions? A meta-analysis and systematic review of randomized, controlled trials

**DOI:** 10.1186/1471-2334-11-181

**Published:** 2011-06-22

**Authors:** Pranita D Tamma, Nirupama Putcha, Yong D Suh, Kyle J Van Arendonk, Michael L Rinke

**Affiliations:** 1 Department of Pediatric Infectious Diseases, Johns Hopkins Medical Institutions, Baltimore, MD, USA; 2 Department of Pulmonary and Critical Care Medicine, Johns Hopkins Medical Institutions, Baltimore, MD, USA; 3 Johns Hopkins Medical Institutions, Baltimore, MD, USA; 4 Department of Surgery, Johns Hopkins Medical Institution, Baltimore, MD, USA; 5 Department of Pediatrics, Johns Hopkins Medical Institutions, Baltimore, MD, USA

**Keywords:** β-lactams, infusion, multi-drug resistant Gram-negatives, antibiotics

## Abstract

**Background:**

The emergence of multi-drug resistant Gram-negatives (MDRGNs) coupled with an alarming scarcity of new antibiotics has forced the optimization of the therapeutic potential of available antibiotics. To exploit the time above the minimum inhibitory concentration mechanism of β-lactams, prolonging their infusion may improve outcomes. The primary objective of this meta-analysis was to determine if prolonged β-lactam infusion resulted in decreased mortality and improved clinical cure compared to intermittent β-lactam infusion.

**Methods:**

Relevant studies were identified from searches of MEDLINE, EMBASE, and CENTRAL. Heterogeneity was assessed qualitatively, in addition to I^2 ^and Chi-square statistics. Pooled relative risks (RR) and 95% confidence intervals (CI) were calculated using Mantel-Haenszel random-effects models.

**Results:**

Fourteen randomized controlled trials (RCTs) were included. Prolonged infusion β-lactams were not associated with decreased mortality (n= 982; RR 0.92; 95% CI:0.61-1.37) or clinical cure (n = 1380; RR 1.00 95% CI:0.94-1.06) compared to intermittent infusions. Subgroup analysis for β-lactam subclasses and equivalent total daily β-lactam doses yielded similar results. Most studies had notable methodological flaws.

**Conclusions:**

No clinical advantage was observed for prolonged infusion β-lactams. The limited number of studies with MDRGNs precluded evaluation of prolonged infusion of β-lactams for this subgroup. A large, multicenter RCT with critically ill patients infected with MDRGNs is needed.

## Background

In the last several years, a progressive increase in resistance among Gram-negative pathogens has continued unabated. The emergence of multi-drug resistant Gram-negative organisms (MDRGNs) coupled with an alarming scarcity of new antibiotic classes in the pipelines of the pharmaceutical industry has forced the healthcare community to optimize the therapeutic potential of currently available antibiotics [1].

The primary determinant of β-lactam efficacy is the duration of time in which the non-protein bound drugconcentration (fT) exceeds the minimum inhibitory concentration (MIC) of the organism (fT>MIC) [2]. β-lactam antibiotics have traditionally been administered by intermittent infusion. With intermittent dosing, β-lactams attain a high peak concentration, but short half-lives can lead to precipitous drops in serum drug levels. Optimizing fT>MIC is particularly difficult for organisms with elevated MICs. Pharmacokinetic studies have shown that prolonging the infusion time provides more consistent serum levels and maximizes fT>MIC [3-7]. It is unclear, however, if this translates to improved patient outcomes.

Several trials comparing clinical outcomes of prolonged infusion β-lactams with standard dosing have been completed, with conflicting results [7]. Moreover, the interpretation of these studies remains controversial as most trials were conducted with small numbers of patients. We performed a systematic review and meta-analysis of randomized controlled trials (RCTs) investigating the efficacy of prolonged infusion β-lactam therapy compared with intermittent infusion β-lactam therapy with regards to mortality, clinical cure, and adverse effects. The primary objective was to determine if prolonged infusion of β-lactam antibiotics resulted in improved patient survival and clinical cure compared to intermittent dosing of β-lactam antibiotics. The secondary objective was to determine if prolonged infusion of β-lactam antibiotics resulted in increased adverse effects compared to standard, bolus dosing of β-lactam antibiotics.

## Methods

### Definitions

Prolonged infusion of β-lactam antibiotics consisted of either extended infusion or continuous infusion of the antibiotic. Extended infusions were defined as intermittent infusions lasting ≥ 3 hours, whereas continuous infusion involved administration over a 24-hour period at a fixed rate [8]. Intermittent infusion of β-lactam antibiotics served as the control group and was defined as standard infusions of antibiotics based on package inserts. Identical β-lactams did not need to be administered for both study arms in a particular trial, so long as both drugs were β-lactam antibiotics with similar spectrums of activity.

### Outcomes

The primary outcomes of the analysis were mortality and clinical cure. Mortality was assumed to be in-hospital mortality, a biologically relevant period in which death can be considered a consequence of treatment failure. Clinical cure was defined by the discretion of the authors because of the heterogeneous nature of the study population, pathogens involved, and the sites of infections. The secondary outcome of the analysis was adverse effects during β-lactam treatment.

### Data sources

Relevant studies were identified from searches of MEDLINE, EMBASE, and Cochrane Central Register of Controlled Trials (CENTRAL) without imposing language or study period restrictions. Databases were searched on February 11, 2011 utilizing the final search strategy. Appendix A contains the complete electronic search strategy. Search terms for MEDLINE included descriptors of population, drug, and administration schedule. A similar method was employed to search EMBASE, with different limits to capture clinical trials of humans and to exclude review articles and case reports. Highly sensitive RCT filters were incorporated for MEDLINE and EMBASE searches. For CENTRAL a strategy similar to that used for MEDLINE was utilized, without the highly sensitive term for clinical trials. In addition, due to the smaller size of the CENTRAL database, some terms in the search strategy were altered to increase the sensitivity of the search. Two translators with a medical background were used for any non-English articles encountered. After a final list of included articles was compiled, Web of Science (accessed March 2^nd^, 2011) and a review of the citations for each article were conducted to search for further potentially relevant articles to include. First authors of studies needing further clarification were contacted [5,9-12].

### Study Selection

Two independent reviewers examined studies for inclusion at both title/abstract and full-text review stages. Any discrepancies between reviewers were resolved by consensus of all study members. During initial title and abstract selection, a broad criterion for inclusion was encouraged and consisted of any study comparing prolonged infusion β-lactams to intermittent β-lactam infusion in humans. Full text review was then conducted with more conservative inclusion requirements. A study was considered eligible if (1) it was an RCT, (2) it compared prolonged infusion β-lactam antibiotics to intermittent β-lactam antibiotics as treatment for hospitalized patients with infections, and (3) it was conducted in humans. Trials focusing on pharmacokinetic or pharmacodynamic parameters with no description of clinical outcomes were excluded. Cross-over RCTs were excluded given concern that this intervention would obscure the benefit of either method of β-lactam administration. Standardized data abstraction forms and risk of bias forms were completed for all included studies.

### Data extraction

Data were extracted by two independent reviewers. Any disagreement was resolved by consensus with the remaining reviewers. The following data were abstracted from each study: study setting and time period, patient ages, baseline APACHE II scores, body sites of infection, and responsible pathogens. Antibiotic name, dose, interval, and duration of infusion, as well as use of additional antibiotics were also recorded. Definition of mortality, clinical cure, adverse effects, and number of participants discontinuing therapy were collected. Extracted data were entered into RevMan version 5.0 software (Copenhagen: The Nordic Cochrane Centre, The Cochrane Collaboration, 2008) and a second author independently confirmed accurate data entry into this program.

### Methodological quality

Two independent reviewers utilized guidelines from the Cochrane Handbook for Systematic Reviews of Interventions to assess manuscript quality [13]. Disagreements were resolved by group consensus. Parameters evaluated included sequence generation, allocation concealment, masking, selective outcome reporting, differential loss to follow-up, and intention-to-treat analysis. The source of the study's funding was also documented. If any of this information could not be determined from study description, "unclear" was documented.

### Data analysis and statistical methods

Statistical analyses were performed using RevMan 5.0 software. After a qualitative assessment of heterogeneity, quantitative heterogeneity was assessed by Chi^- ^square statistics. The extent of the inconsistencies was characterized using the I^2 ^statistic. Considerable heterogeneity was indicated by I^2^>50% [13]. All outcomes were reported as dichotomous. Pooled relative risks (RR) and 95% confidence intervals (CI) for mortality and clinical cure were calculated by use of the Mantel-Haenszel random-effects model given the heterogeneity observed between studies. Publication bias was assessed using funnel plots on mortality and clinical cure endpoints. Sensitivity analyses were performed to test our assumption that inclusion of studies without strict intention-to-treat analysis did not significantly alter results compared to exclusion of these studies. Planned sub-group analyses included the following: continuous infusion β-lactams (excluding extended infusion), β-lactam subclasses, exclusion of pharmaceutical industry funded trials, infections with MDRGNs, and restriction to studies with equivalent total daily dose of β-lactams in both arms. For all analyses, a p-value < 0.05 was regarded as statistically significant.

## Results

### Study selection

**Figure 1:**
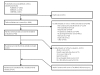
**Flow diagram of selection of articles for inclusion in meta-analysis of randomized controlled trials comparing clinical outcomes of prolonged infusion and intermittent infusion of β-lactams in hospitalized inpatients**.

We identified 3181 potentially relevant published articles on review of MEDLINE (n = 1884), EMBASE (n = 894), and CENTRAL (n = 403) (Figure 1). After removing 181 duplicates, 3000 titles and abstracts were reviewed by two independent study team members. Forty-four studies underwent full text review and 13 met criteria for inclusion. After review of Web of Science and hand searching the citation list of each included study, 1 additional study was included [14]. The baseline characteristics of the 14 included studies are described in Table 1[3,4,9,11,14-23].

**Table 1:** Characteristics of eligible studies included in a meta-analysis of prolonged infusion versus intermittent infusion of β-lactams in hospitalized patients

Study	Country	Setting	Sample Size	Type of Infection	Mean Age (range)	Mean APACHE II Score (range)	Definition of Clinical Cure
Angus 2000	Thailand	Not specified	21	Septicemia, meliodosis	NA (27-73)	NA (3-27)	Not specified

Bodey 1979	USA	Non-ICU	204	Bacteremia, pneumonia, UTI†, neutropenic Fever	Not specified	Not specified	Disappearance of all clinical and laboratory evidence of infection at the time administration of antibiotics was discontinued

Buck 2005	Germany	Non-ICU	24	Various	60.3 (32-88)	Not specified	Improvement of clinical and laboratory signs of infection: resolution of fever, decreased CRP, normalized leukocytes, CXR resolution

Georges 2005	France	ICU	50	Pneumonia, bacteremia	48.0	Not specified	Complete resolution of infectious signs without further need for antibiotics

Hanes 2000	USA	ICU	32	Pneumonia	34.5	11.6	Complete resolution of all signs/symptoms of pneumonia, or improvement in 1+ signs/symptoms of pneumonia

Kojika 2005	Japan	Not specified	10	Abdominal abscesses	63.7 (43-85)	12.5 (9-21)	Afebrile and normalized white blood cell count

Lagast 1983	Belgium	Not specified	45	Septicemia	Not specified	Not specified	Disappearance of all clinical and laboratory evidence of infection

Lau 2006	USA	ICU	262	Abdominal infections	49.8 (18-95)	7.9 (0-31)	Complete resolution of clinical signs and symptoms or improvement (reduction of majority of signs and symptoms and no new signs of infection)

Lubasch 2003	Germany	Not specified	81	COPD†† exacerbations	65.3	Not specified	Recurrence to situation before exacerbation

Merchant 2008	USA, Europe	ICU	531	Pneumonia	51.5	NA (8-29)	Microbiologic and clinical response

Nicolau 2001	USA	ICU	41	Pneumonia, bacteremia	51.1	14.7	Complete resolution of pneumonia or lack of progression of abnormalities on chest radiograph

Rafati 2006	Iran	ICU	40	Pneumonia, bacteremia, UTIs, SSI, abdominal infections	49	15.3	Change in APACHE II score, afebrile, normalization of WBC

Roberts 2007	Australia	ICU	57	Septicemia	47.4	17.6	Disappearance of all signs and symptoms related to the infection

Van Zanten 2006	Netherlan ds	Not specified	93	COPD exacerbations	66.0 (34-76)	Not specified	Infiltrate improvement on x-ray, clinical improvement, and no need for antibiotic treatment within 48 h after cefotaxime discontinuation

†Urinary tract infection; ††chronic obstructive pulmonary disease

### Study characteristics

Study sample sizes varied from 10 to 531 patients. Studies were conducted on at least 4 continents, with the majority in North America or Europe. Year of publication ranged from 1979 to 2008, with all but two studies published after the year 2000 [15,19]. Patients were admitted to the intensive care unit for all or part of their admission in half of the included studies [4,11,16,20,21].

Extended infusion β-lactams were administered in the experimental arm in 3 studies, with the infusion time ranging from 3-7 hours [14,18,21]. The remaining studies included patients receiving continuous infusion β-lactams [3,4,9,11,15-17,19,20,22,23]. Six studies used the same total daily dose of β-lactams in both study arms [9,15,17-20]. Table 2 describes the antibiotic dose and infusion schedules in each study. In all studies utilizing a continuous infusion mechanism of β-lactam delivery, a loading dose of the β-lactam was initially administered to ensure early attainment of fT>MIC. Patients received additional non-β-lactam antibiotics in 11 studies [4,9,11,14-17,19,21,22]. The medication in the study arms differed in one trial in which doripenem was compared to imipenem [21]. Because of the similar spectrum of activity of these agents, this study was included. Duration of therapy varied markedly between studies with a mean of 9.4 days. In several studies, β-lactam dosing was adjusted for renal insufficiency [4,11,16,20,24].

**Table 2:** Antibiotic dosage and outcome data of eligible studies included in a meta-analysis of prolonged infusion versus intermittent infusion of β-lactams in hospitalized patients

		Antibiotic Dose and Infusion Schedule		Number Randomized		Number Analyzed		Adverse Effects		
**Study**	**Antibiotic**	**Intermittent**	**Prolonged**	**Intermittent**	**Prolonged**	**Intermittent**	**Prolonged**	**Definition**	**Intermittent n, %**	**Prolonged n, %**

Angus 2000	Ceftazidime	40 mg/kg q8 h	4 mg/kg/h over 24 h; 12 mg/kg LD over 30 min*	11	10	11	10	Not specified	NA	NA

Bodey 1979	Cefamandole	3 g q6 h	12 g over 24 h; 3 g LD over 30 min	92	74	92	74	Renal failure	17 (18)	14 (19)

Buck 2005	Piperacillin/Tazobactam	4 g/0.5 g q8 h	8 g over 24 h; 2 g LD over 60 min	12	12	12	12	Not specified	NA	NA

Georges 2005	Cefepime	2 g q12 h	2 g q12 h over 12 h	24	26	24	26	Bacterial superinfection	4 (17)	3 (12)

Hanes 2000	Ceftazidime	2 g q8 h	60 mg/kg over 24 h; 2 g LD over 30 min	15	17	14	16	Bacterial superinfection	3 (22)	7 (44)

Kojika 2005	Meropenem	0.5 g q8 h	0.5 g q8 h over 3 h	5	5	5	5	Hepatotoxicity or renal failure	0	0†††

Lagast 1983	Cefoperazone	2 g q12	4 g over 24 h; 1 g LD over 15 min	25	20	25	20	Diarrhea, phlebitis, skin rash	NA	NA

Lau 2006	Piperacillin/Tazobactam	3 g/0.375 g q6 h	12 g/1.5 g over 24 h; 2 g LD over 30 min	132	130	130	128	Gastrointestinal and CNS disturbances	115 (87)	116 (89)

Lubasch 2003	Ceftazidime	2 g q8 h	2 g q12 h over 7 h; 2 g LD over 30 min	40	41	40	41	Gastrointestinal and CNS disturbances	NA	NA

Merchant 2008	Doripenem & Imipenem	Imipenem 500 mg q6 h min;1 g q8 h	Doripenem 500 mg q8 h over 4 h	267	264	252	249	Hepatotoxicity, diarrhea, rash, nausea	21 (8)	24 (10)

Nicolau 2001	Ceftazidime	2 g q8 h	3 g over 24 h; 1 g LD over 30 min	19	22	18	17	Diarrhea, rash	NA	NA

Rafati 2006	Piperacillin	3 g q6 h	8 g over 24 h; 2 g LD over 30 min	20	20	20	20	Not specified	NA	NA

Roberts 2007	Ceftriaxone	2 g q24 h	2 g over 24 h; 0.5 g LD as "bolus"	28	29	28	29	Renal failure	NA	NA

Van Zanten 2006	Cefotaxime	1 g q8 h	2 g over 24 h; 1 g LD over 30 min	46	47	43	40	Not specified	NA	NA

*LD - loading dose

### Risk of bias within studies

In general, there were few methodologically sound studies with adequate sample sizes to definitively determine whether prolonged infusion β-lactams are superior to intermittent infusion β-lactams for the treatment of infections in hospitalized patients. As outlined in Table 3, all included studies were RCTs but the method of randomization was detailed in only three trials [9,11,15]. Inclusion and exclusion criteria were clearly defined in all trials. All studies included participants ≥18 years of age, although Angus et al and Hanes et al had younger age limits of 14 years and 16 years, respectively [3,4]. Allocation concealment was implemented in only two studies, as seen in Table 2[9,15]. The remaining studies largely failed to address allocation concealment. Masking was conducted in only one study [9]. Intention-to-treat analysis was conducted in eight studies [9,15-19,21,22]. A sensitivity analysis was conducted including only those studies that clearly indicated intention-to-treat analysis to investigate the impact of decreasing methodological quality among studies on our pooled RRs. The quantitative summary measure of effect remained largely unchanged, suggesting that our results were not disproportionately influenced by inclusion of studies not utilizing intention-to-treat analyses (Table 4).

**Table 3:** Risk of bias assessment of eligible studies included in a meta-analysis of prolonged infusion versus intermittent infusion of β-lactams in hospitalized patients

Study	Funding Source	Allocation Sequence Adequately Generated	Allocation Concealment	Masking	Similar Rates of Withdrawals Between Groups	Intention to Treat Analysis
Angus 2000	Wellcome Trust of Great Britain	Unclear	Unclear	Unclear	Yes	No

Bodey 1979	Pharmaceutical company	Yes	Yes	Unclear	Unclear	Yes

Buck 2005	Pharmaceutical company	Unclear	Unclear	No	Yes	Yes

Georges 2005	Not specified	Unclear	Unclear	No	Yes	Yes

Hanes 2000	Pharmaceutical company	Unclear	Unclear	Unclear	Yes	No

Kojika 2005	Not specified	Unclear	No	No	Yes	Yes

Lagast 1983	Pharmaceutical company	Unclear	Unclear	Unclear	Yes	Yes

Lau 2006	Pharmaceutical company	Unclear	Unclear	No	Yes	No

Lubasch 2003	Pharmaceutical company	Unclear	Unclear	No	Unclear	Unclear

Merchant 2008	Pharmaceutical company	Unclear	Unclear	No	Yes	Yes

Nicolau 2001	Pharmaceutical company	Yes	Unclear	No	Yes	No

Rafati 2006	Tehran University Medical Sciences Research Board	Unclear	Unclear	No	Yes	Yes

Roberts 2007	National Health & Medical Research Council	Yes	Yes	Yes^a^	Yes	Yes

Van Zanten 2006	Pharmaceutical company	Unclear	Unclear	No	No	No

^a^Only outcome assessors were masked

**Table 4:** Summary of subgroup and sensitivity analysis of eligible studies included in a meta-analysis of prolonged infusion versus intermittent infusion of β-lactams in hospitalized patients†

Sub-group analysis	Studies Included	Mortality Risk Ratio (95% CI)	I^2 ^%	Studies Included	Clinical Cure Risk Ratio (95% CI)	I^2^%
β-lactam subclasses						
Penicillins	2	0.62 (0.19-2.03)	0	3	0.77 (0.46-1.30)	0
Cephalosporins	4	0.95 (0.35-2.63)	50	8	1.04 (0.92-1.18)	35
Carbapenems	2	1.08 (0.64-1.82)	0	2	1.00 (0.69-1.44)	0
Continuous infusion	6	0.80 (0.42-1.50)	22	10	1.01 (0.92-1.10)	16
Not funded by pharmaceutical industry	5	0.80 (0.37-1.73)	26	5	1.15 (0.85-1.57)	57
Equivalent daily dose of β-lactam antibiotic	5	1.30 (0.59-2.87)	0	6	1.06 (0.90-1.25)	48
**Sensitivity-analysis**						
Intention-to-treat analysis	8	1.10 (0.75-1.60)	0	8	1.05 (0.93-1.19)	21

† Reference group is intermittent β-lactam infusion

**Figure 2:**
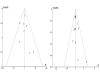
**Funnel plots demonstrating the possibility of a small publication bias assessing studies reporting mortality (left) but low probability of publication bias assessing studies reporting clinical cure (right) comparing prolonged and intermittent infusion of β-lactam antibiotics in hospitalized patients**.

A pharmaceutical company was identified as the source of funding for nine trials [3,11,14-16,19-21,23]. Funnel plots comparing prolonged infusion and intermittent infusion β-lactams were performed to screen for evidence of publication bias for the mortality and clinical cure outcomes (Figure 2). For the mortality outcome, the plot suggested a paucity of studies indicating a protective effect of prolonged β-lactam infusion of mortality. A funnel plot evaluating the outcome of clinical cure, however, was relatively symmetrical, suggesting minimal publication bias. Overall, based on qualitative and quantitative exploration, no conclusive evidence of reporting bias was found.

### Mortality

**Figure 3:**
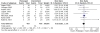
**Mortality comparing prolonged infusion and intermittent infusion of β-lactam antibiotics in hospitalized patients**.

Eight studies reported mortality as an outcome (Figure 3). Among the 487 patients enrolled in the prolonged infusion β-lactam arm, there were 53 deaths, compared to 56 deaths among the 495 patients in the intermittent infusion arm. These differences were not statistically significant with an RR of 0.92 (95% CI 0.61 - 1.37). The overall I^2 ^statistic was 9% suggesting relatively low heterogeneity between the studies. Similarly, the Chi-square statistic was 7.66, p = 0.36. All studies except one crossed the null value [4]. Mortality ranged from 2% in a trial consisting of a relatively young population with low severity of illness to 57% in a severely ill population infected with a highly pathogenic organism, *Burkholderia pseudomallei *[4,20]. Only one study demonstrated a mortality advantage to prolonged infusion β-lactams [4]. This was the only study in which all included subjects were in critical condition and had bacterial cultures confirming infection with a resistant Gram-negative organism.

Because of an *a priori *hypothesis that certain β-lactams may be more effective with prolonged infusion mechanisms than others, subgroup analysis of each of the β-lactam subclasses was conducted. This hypothesis was based on the observation that some β-lactams, like piperacillin, have a MIC breakpoint higher than postulated to be effective based on Monte Carlo simulation techniques [8]. No significant differences were found between subclasses, although each of these subgroups was relatively small. Similarly, subgroup analyses including only studies that used continuous infusion β-lactams, only non-pharmaceutical industry sponsored trials, and only studies utilizing equivalent total daily β-lactam dose in both study arms did not yield results different from the pooled RR derived from inclusion of all studies (Table 4). A subgroup analysis was planned for studies with highly resistant organisms, but the MIC for organisms was not reported in the vast majority of trials, precluding completion of this subgroup analysis.

### Clinical cure

**Figure 4:**
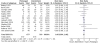
**Clinical cure comparing prolonged infusion and intermittent infusion of β-lactam antibiotics in hospitalized patients**.

All but one study included clinical cure as an outcome (Figure 4) [4]. Proportion of clinical cure ranged from 32% to 100%. In the prolonged arm, 470 out of 677 evaluable patients were considered clinical successes. This compared to 479 out of 703 in the standard infusion arm. The pooled RR was 1.00 (95% CI 0.94-1.06). The I^2 ^statistic was 0%, and similarly the Chi-square statistic did not indicate heterogeneity (p = 0.58). Similar to mortality, no significant difference was observed in clinical success between prolonged infusion and intermittent infusion β-lactam antibiotics. Only one study demonstrated a statistically significant clinical cure outcome, favoring the prolonged infusion arm [9]. Of importance, this was the only study in which the allocation sequence was adequately generated, allocation concealment was appropriate, some degree of blinding occurred, and appropriate intention to treat analysis was utilized. Subgroups for clinical cure were analyzed in the same manner as mortality, with results that were similar to pooled estimates as displayed in Table 4.

### Adverse effects

**Figure 5:**
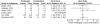
**Adverse effects comparing prolonged and intermittent infusion of β-lactam antibiotics in hospitalized patients**.

Six studies reported adverse effects during administration of the study medication. The most common reported adverse effects were diarrhea and hepatotoxicity. For almost all studies, the inclusion criteria for adverse effects were not developed *a priori*. The definition of adverse effects varied substantially between studies preventing calculation of a meaningful pooled RR ratio (Figure 5).

### Study withdrawals

**Figure 6:**
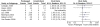
**Study participant withdrawals comparing prolonged and intermittent infusion of β-lactam antibiotics in hospitalized patients**.

Differential losses to follow-up appeared to be minimal, as rates of study withdrawals were relatively similar between study arms (Figure 6). No study reported greater than 12% study withdrawal. [3,4,9,16-22,24] Because of the scarcity of details regarding reasons for withdrawals, a summary statistic for withdrawals was not calculated.

## Discussion

Several observational studies with varying study designs comparing clinical benefits of prolonged and intermittent infusion of β-lactam antibiotics have been conducted with inconsistent results. A prospective study of 98 hospitalized patients who were prescribed piperacillin-tazobactam revealed a trend towards greater clinical success in the continuous infusion group when compared with intermittent infusion recipients [25]. In a retrospective cohort study of piperacillin-tazobactam for *Pseudomonas aeruginosa *sepsis, a significantly lower 14-day mortality rate and shorter hospital length of stay in patients receiving extended infusion, was demonstrated [26]. Results from another retrospective cohort study showed increased clinical cure by continuous versus intermittent infusion of piperacillin-tazobactam in adults with ventilator-associated pneumonia (VAP) caused by Gram-negative pathogens with MICs ≥8 μg/ml [27]. A third retrospective cohort study, however, failed to demonstrate improved clinical outcomes with extended infusion piperacillin-tazobactam [28]. Drug-related adverse effects were mild and reported in similar numbers in both treatment arms in all of these studies [25-28]. Retrospective studies comparing continuous infusion versus intermittent infusion cefepime and meropenem in patients with VAP both noted significantly improved clinical cure rates in the continuous infusion arms [29,30].

In an attempt to clarify the comparative effect of prolonged versus intermittent infusion β-lactam antibiotics on mortality, clinical cure, and adverse effects, we analyzed RCTs that offered the most methodologically rigorous evidence from the available studies. Our results do not demonstrate a clinical advantage to prolonged infusion β-lactams for routine use in hospitalized patients. Sensitivity analyses and various sub-group analyses determined by *a priori *biologically-plausible hypotheses similarly did not indicate an advantage to prolonged infusion β-lactams.

Two previous systematic reviews and meta-analyses were published to answer similar questions [31,32]. A previous meta-analysis included continuous infusion of aminoglycosides and vancomycin, making it difficult to isolate the benefit of β-lactams [32]. A prior systematic review focused on continuous infusion β-lactams in the prolonged arm and included cross-over studies [31]. *In vitro *evidence demonstrates similar properties of fT>MIC for both extended and continuous infusion β-lactams making their effect likely comparable, thus we elected to include both groups under the umbrella term "prolonged infusion," a decision that was further supported by our sub-group analysis of only studies examining continuous β-lactam infusions. Inclusion of cross-over studies obscures clinical benefits attributable to the different infusion schedules, and therefore these studies were excluded in the present review. The current meta-analysis includes two additional RCTs, consisting of 612 patients, compared with those published previously [14,21]. Overall, the findings of our meta-analysis are consistent with those previously described [31,32].

Our study has several limitations that should be taken into account when interpreting the results. Overall, the included studies were of moderate to poor quality with regards to answering our clinical question of interest and had notable methodological differences. Allocation sequence generation and allocation concealment were not addressed in most studies. As a result, most of these studies were at risk for selection bias. Similarly, most of the studies did not adequately address masking, making detection bias a consideration. Although masking of health care providers and patients may be difficult to impose because of the nature of the intervention, masking of outcome assessors would certainly be reasonable. Of interest, the study with the most stringent methods was the only one to show a clinical cure advantage for prolonging β-lactam infusions [9]. Most trials were funded by the pharmaceutical industry, making reporting bias a concern. Studies conducted by the pharmaceutical industry with unfavorable results for prolonged infusion β-lactams may be less likely to be published [33].

Another noteworthy limitation is that participants in the available studies were frequently receiving additional antibiotics, generally aminoglycosides, limiting conclusions about the sole contribution of the β-lactam antibiotics. In reality, however, when prolonged β-lactams are administered, it is generally a final attempt to rid the body of a particular pathogen and is administered in conjunction with aminoglycoside therapy, making the design of several of these studies similar to actual clinical practice [7]. Only six of the studies used the same total daily dose of β-lactam antibiotic in both arms [8,9,15,17-19]. The majority of studies used a higher dose of antibiotic in the intermittent infusion arm and this unequal treatment favoring standard infusions could have biased the results towards the null. Subgroup analysis of studies with equivalent total daily dose of β-lactam antibiotic in both arms was conducted and did not demonstrate any significant results; however, interpretation of these results is limited by the relatively small sample size of this population and the notable heterogeneity of these trials (I^2 ^=50%).

Perhaps the biggest limitation to our study is that the bacterial isolates identified in the included RCTs generally had MICs in the susceptible range. Little difference exists between intermittent and prolonged administration of β-lactams in achieving fT>MIC in this range of susceptibility [2]. However, when less susceptible organisms are present, the likelihood for treatment failure increases with intermittent dosing. In one study, maintaining a fT>MIC of 100% for cephalosporins was associated with significantly improved clinical cure compared to maintenance of fT>MIC at lower percentages [34]. Prolonged infusion therapy is generally considered in clinical scenarios involving MDRGN organisms with elevated MICs. From the available RCTs, it cannot be determined if prolonged infusion β-lactams result in greater clinical success than standard β-lactam infusions when MDRGNs are the offending pathogens. Interestingly, the one study conducted in critically ill patients with microbiological evidence of a resistant Gram-negative organism did in fact show a mortality benefit in favor of prolonged β-lactam administration [4]. Perhaps, inclusion of studies with a low burden of disease and highly susceptible microorganisms in our review may have diluted the true effect of prolonged antibiotic infusion regimens.

Despite these limitations, this systematic review and meta-analysis add useful information to the literature. The 14 included studies span 4 continents and are not restricted to the English language, thus increasing generalizability. As only RCTs were included, baseline characteristics including age, severity of illness, body site of infection, responsible pathogens, and underlying medical conditions were similar between the treatment groups. Confounding by indication can be extremely problematic in observational studies, as prognostic factors may influence treatment decisions. However, this was unlikely in the included trials because of the process of randomization.

Optimizing the pharmacokinetics-pharmacodynamics of currently available antibiotics is necessary in the era of MDRGN infections. Known β-lactam pharmacodynamics support the concept of prolonged infusion to maximize fT>MIC, but limited clinical data exist regarding the comparative efficacy of prolonged β-lactam infusion. The results of this meta-analysis do not support an advantage to the use of prolonged infusion β-lactam antibiotics as standard practice for hospitalized patients. These findings remained consistent in the multiple sensitivity and subgroup analyses that were evaluated.

## Conclusions

Prolonged infusion β-lactam antibiotics may have value in a specific subsets of patients, such as those with highly-resistant Gram-negative infections because of exploitation of their property of fT>MIC. Unfortunately, the very limited number of patients in the included RCTs with MDRGNs precluded evaluation of this subgroup in the present meta-analysis. Methodologically rigorous studies analyzing prolonged infusion β-lactams for critically ill patients with MDRGN infections are necessary to substantiate this potential benefit.

## List of abbreviations used

MDRGNs: multidrug-resistant Gram-negatives; MIC: minimum inhibitory concentration; RCT: randomized controlled trial; CENTRAL: Cochrane Central Register of Controlled Trials; RR: relative risk; CI: confidence interval; VAP: ventilator-associated pneumonia;

## Competing interests

The authors declare that they have no competing interests.

## Authors' contributions

PDT formulated the research question and was involved in manuscript preparation. All authors were involved in determining the search strategy, data collection for the review, and quantitative and qualitative analysis. All authors read and approved the final manuscript.

## Endnotes

### APPENDIX: Final Electronic Search Strategy (February 11, 2011)

#### MEDLINE Search Strategy

(("beta-lactams"[MeSH] OR "beta-lactams"[tiab] OR "beta lactam"[tiab]) AND ("anti-bacterial agents"[MeSH] OR ("anti-bacterial"[tiab] AND "agents"[tiab]) OR "anti-bacterial agents"[tiab] OR "antibiotics"[tiab] OR "antibiotic"[tiab]) OR "beta-lactam antibiotics"[tiab] OR "beta-lactam antibiotic"[tiab] OR doripenem[Title/Abstract] OR cefepime[Title/Abstract] OR ceftazidime[Title/Abstract] OR piperacillin[Title/Abstract] OR piperacillin/tazobactam[tiab] OR piperacillin-tazobactam[tiab] OR cefamandole[tiab] OR cefazolin[tiab] OR cefotaxime[tiab] OR ceftriaxone[tiab] OR imipenem[tiab] OR meropenem[tiab] OR ertapenem[tiab] OR cefoperazone[tiab] OR penicillin[tiab] OR penicillins[tiab] OR imipenem-cilastatin[tiab] OR ampicillin[tiab] OR ampicillin-sulbactam[tiab] OR sulbactam-ampicillin[tiab] OR tazocin[tiab] OR carbenicillin[tiab] OR carbapenem[tiab] OR cephalosporin[tiab] OR cephalosporins[tiab] OR ticarcillin-clavulanate[tiab] OR cefpirome[tiab] OR flucloxacillin[tiab] OR mezlocillin[tiab] OR aztreonam[tiab] OR cefuroxime[tiab] OR ceftizoxime[tiab])

AND

("Drug Administration Schedule"[Mesh] OR "Infusions, Intravenous"[MeSH] OR "continuous infusion"[Title/Abstract] OR "extended infusion"[Title/Abstract] OR "intermittent therapy"[Title/Abstract] OR ((continuous[tiab] OR bolus[tiab] OR extended[tiab] OR intermittent[tiab]) AND (administration[tiab] OR infusion[tiab] OR dosing[tiab])))

AND

("hospitalized"[tiab] OR "hospitalised"[tiab] OR "hospitalization"[tiab] OR "hospitalisation"[tiab] OR "Bacterial Infections"[Mesh] OR "sepsis"[Title/Abstract] OR (("intensive"[tiab] OR "critical"[tiab] OR "acute"[tiab]) AND ("care"[tiab] OR "unit"[tiab] OR "illness"[tiab])))

AND

(randomized controlled trial[pt] OR controlled clinical trial[pt] OR randomized[tiab] OR placebo[tiab] OR drug therapy[sh] OR randomly[tiab] OR trial[tiab] OR groups[tiab] NOT (animals[mh] NOT humans[mh]))

#### Cochrane Search Strategy

(("beta-lactams"[MeSH] OR "beta-lactams"[all fields] OR "beta lactam"[all fields]) AND ("anti-bacterial agents"[MeSH] OR ("anti-bacterial"[all fields] AND "agents"[all fields]) OR "anti-bacterial agents"[all fields] OR "antibiotics"[all fields] OR "antibiotic"[all fields]) OR "beta-lactam antibiotics"[tiab] OR "beta-lactam antibiotic"[tiab] OR doripenem[Title/Abstract] OR cefepime[Title/Abstract] OR ceftazidime[Title/Abstract] OR piperacillin[Title/Abstract] OR piperacillin/tazobactam[tiab] OR piperacillin-tazobactam[tiab] OR cefamandole[tiab] OR cefazolin[tiab] OR cefotaxime[tiab] OR ceftriaxone[tiab] OR imipenem[tiab] OR meropenem[tiab] OR ertapenem[tiab] OR cefoperazone[tiab] OR penicillin[tiab] OR penicillins[tiab] OR imipenem-cilastatin[tiab] OR ampicillin[tiab] OR ampicillin-sulbactam[tiab] OR sulbactam-ampicillin[tiab] OR tazocin[tiab] OR carbenicillin[tiab] OR carbapenem[tiab] OR cephalosporin[tiab] OR cephalosporins[tiab] OR ticarcillin-clavulanate[tiab] OR cefpirome[tiab] OR flucloxacillin[tiab] OR mezlocillin[tiab] OR aztreonam[tiab] OR cefuroxime[tiab] OR ceftizoxime[tiab])

AND

("Drug Administration Schedule"[Mesh] OR "Infusions, Intravenous"[MeSH] OR "continuous infusion"[Title/Abstract] OR "extended infusion"[Title/Abstract] OR "intermittent therapy"[Title/Abstract] OR ((continuous[tiab] OR bolus[tiab] OR extended[tiab] OR intermittent[tiab]) AND (administration[tiab] OR infusion[tiab] OR dosing[tiab])))

AND

("inpatient"[mesh] OR "hospitalized"[tiab] OR "hospitalised"[tiab] OR "hospitalization"[tiab] OR "hospitalisation"[tiab] OR "Bacterial Infections"[Mesh] OR "sepsis"[Title/Abstract] OR (("intensive"[tiab] OR "critical"[tiab] OR "acute"[tiab]) AND ("care"[tiab] OR "unit"[tiab] OR "illness"[tiab])))

#### EMBASE Search Strategy

('hospital patients':ab,ti OR 'hospitalized patients':ab,ti OR 'hospitalised patients':ab,ti OR 'hospital infection'/exp OR ('sepsis':ab,ti) OR ('septicemia':ab,ti) OR ('septic shock':ab,ti) OR ('bacterial infection'/exp) OR ('critically ill patient':ab,ti) OR ('critically ill patients':ab,ti) OR ('critical illness':ab,ti) OR ('systemic inflammatory response syndrome':ab,ti))

AND

(('beta lactams'/exp) OR 'doripenem':ab,ti OR 'cefepime':ab,ti OR 'ceftazidime':ab,ti OR 'piperacillin':ab,ti OR 'piperacillin tazobactam':ab,ti OR 'piperacillin':ab,ti OR 'piperacillin tazobactam combination product':ab,ti OR 'tazobactam':ab,ti OR 'cefamandole':ab,ti OR 'cefazolin':ab,ti OR 'cefotaxime':ab,ti OR 'ceftriaxone':ab,ti OR 'imipenem':ab,ti OR 'meropenem':ab,ti OR 'ertapenem':ab,ti OR 'cefoperazone':ab,ti OR 'penicillin':ab,ti OR 'penicillins':ab,ti OR 'imipenem-cilastatin':ab,ti OR 'ampicillin':ab,ti OR 'ampicillin-sulbactam':ab,ti OR 'sulbactam-ampicillin':ab,ti OR 'tazocin':ab,ti OR 'carbenicillin':ab,ti OR 'carbapenem':ab,ti OR 'cephalosporin':ab,ti OR 'cephalosporins':ab,ti OR 'ticarcillin-clavulanate':ab,ti OR 'cefpirome':ab,ti OR 'flucloxacillin':ab,ti OR 'mezlocillin':ab,ti OR 'aztreonam':ab,ti OR 'cefuroxime':ab,ti OR 'ceftizoxime':ab,ti)

AND

('drug administration'/exp OR 'intravenous drug administration'/exp OR 'drug intermittent therapy'/exp OR ('continuous infusion':ab,ti) OR ('extended infusion':ab,ti) OR 'intermittent therapy':ab,ti OR (('continuous':ab,ti OR 'bolus':ab,ti OR 'extended':ab,ti OR 'intermittent':ab,ti) AND ('administration':ab,ti OR 'infusion':ab,ti OR 'dosing':ab,ti)))

AND

((('randomized controlled trial'/exp OR 'clinical trial'/exp) OR ('randomization'/exp)))

AND

[humans]/lim

AND

NOT ('review'/exp OR review)

## Acknowledgements and Funding

The authors would like to thank Swaroop Vedula, Dolly Chang, Tianjing Li, and Kay Dickersin for all of their guidance and thoughtful suggestions. We would also like to thank our translators - Haruhi and Tsuyoshi Inokuchi (Japanese) and Lila Bouadma and Patrice Savard (French). We appreciate the assistance of the Welch medical librarians in finalizing our search strategy. P.D.T, A.L.R, and K.J.V. are funded by a National Institute of Health 5KL2RR025006 award. None of the authors report any relevant conflicts of interest.

## Pre-publication history

The pre-publication history for this paper can be accessed here:

http://www.biomedcentral.com/1471-2334/11/181/prepub

## References

[B1] Rice LB The clinical consequences of antimicrobial resistance Curr Opin Microbiol 2009 12 5 476 81 10.1016/j.mib.2009.08.001 19716760

[B2] Drusano GL Antimicrobial pharmacodynamics: critical interactions of 'bug and drug' Nat Rev Microbiol 2004 2 4 289 300 10.1038/nrmicro862 15031728

[B3] Hanes SD Wood GC Herring V Croce MA Fabian TC Pritchard E Boucher BA Intermittent and continuous ceftazidime infusion for critically ill trauma patients Am J Surg 2000 179 6 436 40 10.1016/S0002-9610(00)00388-3 11004326

[B4] Angus BJ Smith MD Suputtamongkol Y Mattie H Walsh AL Wuthiekanun V Chaowagul W White NJ Pharmacokinetic-pharmacodynamic evaluation of ceftazidime continuous infusion vs intermittent bolus injection in septicaemic melioidosis Br J Clin Pharmacol 2000 50 2 184 91 10.1111/j.1365-2125.2000.00179.x 10930972 PMC2014399

[B5] Buijk SL Gyssens IC Mouton JW Van Vliet A Verbrugh HA Bruining HA Pharmacokinetics of ceftazidime in serum and peritoneal exudate during continuous versus intermittent administration to patients with severe intra-abdominal infections J Antimicrob Chemother 2002 49 1 121 8 10.1093/jac/49.1.121 11751775

[B6] McNabb JJ Nightingale CH Quintiliani R Nicolau DP Cost-effectiveness of ceftazidime by continuous infusion versus intermittent infusion for nosocomial pneumonia Pharmacotherapy 2001 21 5 549 55 10.1592/phco.21.6.549.34539 11349744

[B7] Tamma PD Jenh A Milstone AM Prolonged B-lactam Infusion for Gram-negative Infections Pediatr Infect Dis J 2011 30 4 336 7 21407037 10.1097/INF.0b013e31820ef3e5

[B8] Courter JD Kuti JL Girotto JE Nicolau DP Optimizing bactericidal exposure for beta-lactams using prolonged and continuous infusions in the pediatric population Pediatr Blood Cancer 2009 53 3 379 85 10.1002/pbc.22051 19422028

[B9] Roberts JA Boots R Rickard CM Thomas P Quinn J Roberts DM Richards B Lipman J Is continuous infusion ceftriaxone better than once-a-day dosing in intensive care? A randomized controlled pilot study J Antimicrob Chemother 2007 59 2 285 91 17135183 10.1093/jac/dkl478

[B10] Sakka SG Glauner AK Bulitta JB Kinzig-Schippers M Pfister W Drusano GL Sorgel F Population pharmacokinetics and pharmacodynamics of continuous versus short-term infusion of imipenem-cilastatin in critically ill patients in a randomized, controlled trial Antimicrob Agents Chemother 2007 51 9 3304 10 10.1128/AAC.01318-06 17620371 PMC2043189

[B11] Nicolau DP McNabb J Lacy MK Quintiliani R Nightingale CH Continuous versus intermittent administration of ceftazidime in intensive care unit patients with nosocomial pneumonia Int J Antimicrob Agents 2001 17 6 497 504 10.1016/S0924-8579(01)00329-6 11397621

[B12] Pedeboscq S Dubau B Frappier S Hernandez V Veyssieres D Winnock S Pometan JP [Comparison of 2 administration protocols (continuous or discontinuous) of a time-dependent antibiotic, Tazocin] Pathol Biol (Paris) 2001 49 7 540 7 10.1016/s0369-8114(01)00210-3 11642016

[B13] Lefebvre C Manheimer E Glanville J Higgins JPT, Green S Chapter 6: Searching for studies Cochrane Handbook for Systematic Reviews of Interventions Version 5.0.2 (updated September 2009) 2009 The Cochrane Collaboration http://www.cochrane-handbook.org

[B14] Lubasch A Luck S Lode H Mauch H Lorenz J Bolcskei P Welte T Optimizing ceftazidime pharmacodynamics in patients with acute exacerbation of severe chronic bronchitis J Antimicrob Chemother 2003 51 3 659 64 10.1093/jac/dkg111 12615868

[B15] Bodey GP Ketchel SJ Rodriguez V A randomized study of carbenicillin plus cefamandole or tobramycin in the treatment of febrile episodes in cancer patients Am J Med 1979 67 4 608 16 10.1016/0002-9343(79)90242-0 495630

[B16] Buck C Bertram N Ackermann T Sauerbruch T Derendorf H Paar WD Pharmacokinetics of piperacillin-tazobactam: intermittent dosing versus continuous infusion Int J Antimicrob Agents 2005 25 1 62 7 10.1016/j.ijantimicag.2004.08.012 15620828

[B17] Georges B Conil JM Cougot P Decun JF Archambaud M Seguin T Chabanon G Virenque C Houin G Saivin S Cefepime in critically ill patients: continuous infusion vs. an intermittent dosing regimen Int J Clin Pharmacol Ther 2005 43 8 360 9 16119511 10.5414/cpp43360

[B18] Kojika M Sato N Hakozaki M Suzuki Y Takahasi G Endo S Suzuki K Wakabayasi G [A preliminary study of the administration of carbapenem antibiotics in sepsis patients on the basis of the administration time] Jpn J Antibiot 2005 58 5 452 7 16379157

[B19] Lagast H Meunier-Carpentier F Klastersky J Treatment of gram-negative bacillary septicemia with cefoperazone Eur J Clin Microbiol 1983 2 6 554 8 10.1007/BF02016564 6667681

[B20] Lau WK Mercer D Itani KM Nicolau DP Kuti JL Mansfield D Dana A Randomized, open-label, comparative study of piperacillin-tazobactam administered by continuous infusion versus intermittent infusion for treatment of hospitalized patients with complicated intra-abdominal infection Antimicrob Agents Chemother 2006 50 11 3556 61 10.1128/AAC.00329-06 16940077 PMC1635208

[B21] Merchant S Gast C Nathwani D Lee M Quintana A Ketter N Friedland I Ingham M Hospital resource utilization with doripenem versus imipenem in the treatment of ventilator-associated pneumonia Clin Ther 2008 30 4 717 33 10.1016/j.clinthera.2008.04.001 18498921

[B22] Rafati MR Rouini MR Mojtahedzadeh M Najafi A Tavakoli H Gholami K Fazeli MR Clinical efficacy of continuous infusion of piperacillin compared with intermittent dosing in septic critically ill patients Int J Antimicrob Agents 2006 28 2 122 7 10.1016/j.ijantimicag.2006.02.020 16815689

[B23] van Zanten AR Oudijk M Nohlmans-Paulssen MK van der Meer YG Girbes AR Polderman KH Continuous vs. intermittent cefotaxime administration in patients with chronic obstructive pulmonary disease and respiratory tract infections: pharmacokinetics/pharmacodynamics, bacterial susceptibility and clinical efficacy Br J Clin Pharmacol 2007 63 1 100 9 10.1111/j.1365-2125.2006.02730.x 16869814 PMC2000713

[B24] Chastre J Wunderink R Prokocimer P Lee M Kaniga K Friedland I Efficacy and safety of intravenous infusion of doripenem versus imipenem in ventilator-associated pneumonia: a multicenter, randomized study Crit Care Med 2008 36 4 1089 96 10.1097/CCM.0b013e3181691b99 18379232

[B25] Grant EM Kuti JL Nicolau DP Nightingale C Quintiliani R Clinical efficacy and pharmacoeconomics of a continuous-infusion piperacillin-tazobactam program in a large community teaching hospital Pharmacotherapy 2002 22 4 471 83 10.1592/phco.22.7.471.33665 11939682

[B26] Lodise TP Jr Lomaestro B Drusano GL Piperacillin-tazobactam for Pseudomonas aeruginosa infection: clinical implications of an extended-infusion dosing strategy Clin Infect Dis 2007 44 3 357 63 10.1086/510590 17205441

[B27] Lorente L Jimenez A Martin MM Iribarren JL Jimenez JJ Mora ML Clinical cure of ventilator - associated pneumonia treated with piperacillin/tazobactam administered by continuous or intermittent infusion Int J Antimicrob Agents 2009 33 5 464 8 10.1016/j.ijantimicag.2008.10.025 19150225

[B28] Patel GW Patel N Lat A Trombley K Enbawe S Manor K Smith R Lodise TP Outcomes of extended infusion piperacillin/tazobactam for documented Gram-negative infections Diagn Microbiol Infect Dis 2009 64 2 236 40 10.1016/j.diagmicrobio.2009.03.002 19500529

[B29] Lorente L Jimenez A Palmero S Jimenez JJ Iribarren JL Santana M Martin MM Mora ML Comparison of clinical cure rates in adults with ventilator-associated pneumonia treated with intravenous ceftazidime administered by continuous or intermittent infusion: a retrospective, nonrandomized, open-label, historical chart review Clin Ther 2007 29 11 2433 9 10.1016/j.clinthera.2007.11.003 18158083

[B30] Lorente L Lorenzo L Martin MM Jimenez A Mora ML Meropenem by continuous versus intermittent infusion in ventilator-associated pneumonia due to gram-negative bacilli Ann Pharmacother 2006 40 2 219 23 10.1345/aph.1G467 16449546

[B31] Roberts JA Webb S Paterson D Ho KM Lipman J A systematic review on clinical benefits of continuous administration of beta-lactam antibiotics Crit Care Med 2009 37 6 2071 8 10.1097/CCM.0b013e3181a0054d 19384201

[B32] Kasiakou SK Sermaides GJ Michalopoulos A Soteriades ES Falagas ME Continuous versus intermittent intravenous administration of antibiotics: a meta-analysis of randomised controlled trials Lancet Infect Dis 2005 5 9 581 9 10.1016/S1473-3099(05)70218-8 16122681

[B33] Bourgeois FT Murthy S Mandl KD Outcome reporting among drug trials registered in ClinicalTrials.gov Ann Intern Med 2010 153 3 158 66 20679560 10.1059/0003-4819-153-3-201008030-00006 PMC3374868

[B34] Mckinnon PS Paladino JA Schentag JJ Evaluation of area under the inhibitory curve (AUIC) and time above the minimum inhibitory concentration (T>MIC) as predictors of outcome for cefepime and ceftazidime in serious bacterial infections Int J Antimicrob Agents 2008 31 4 345 51 10.1016/j.ijantimicag.2007.12.009 18313273

